# How COVID-19 pandemic restrictions might have led to a more promising year throughout a positive thinking and a good night sleep?

**DOI:** 10.5935/1984-0063.20210001

**Published:** 2021

**Authors:** Miguel Meira e Cruz

**Affiliations:** 1 Centro Cardiovascular da Universidade de Lisboa, Lisbon School of Medicine, Sleep Unit - Lisbon - Lisbon - Portugal.; 2 Faculdade São Leopoldo Mandic, Neuroimmune Pain Interface Lab - Campinas - São Paulo - Brazil.; 3 International Center of Clinical Sleep Medicine and Research, Escola Bahiana de Medicina e Saúde Pública - Salvador - Bahia - Brasil.; 4 Centro Europeu do Sono, Lisbon - Portugal.

Positive thinking is an important emotional resource leading to better face life adversities and to maintain a good mental health either during acute stressful events or lifetime challenges. The difficult times of COVID-19 pandemic have moved the way we understand the world itself as well as associated populational dynamics. Those changes implied ability and resilience from the individual point of view but also from the social and community perspectives. However, as individuals have different vulnerabilities^1^ and distinct degrees of tolerance^2^, the impact of adversities in any of those factors, would be dependent of particular characteristics eventually associated to specific phenotypes and the existence of pathological conditions. Nevertheless, those peculiarities will mostly rely in common psycho-functional matrixes and therefore will have a common physiological basis. Yet, several restrictive critical measures which were imposed by health policies during the COVID-19 pandemic, impacted on people life’s, increasing the probability of behaviorally guided psychosocial dysfunctional patterns associated to stress^3^.

Our team, as other authors, have shown that sleep has been affected worldwide during these times^4^, which has been attributed to different aspects mainly regarding to the lack of exposure to sun light and lack of exercise but also to the changes of daily routines and schedules, online working, and increased psychosocial stress (e.g., anxiety, depression, sensation of injustice, etc.) leading to the disruption of the circadian timing system and sleep. Arguably, positive thinking would contribute to a better resilience allowing better facing and managing the stress of some political, economical, social, and health-related constraints taking part of the current pandemic crisis. Also, arguably, positive thinking could, either directly (e.g., by reducing sleep threshold) and indirectly (e.g., by modulating the stress responses evoked by the above-mentioned factors) impact sleep.

An appreciable amount of behavioral and neurobiological evidence has linked sleep and emotional health. Hence, a particular mediation role was recently stablished between prior-night sleep, current-day events, and positive affect^5^. This relationship is observed in adult and pediatric ages and have been also associated to some chronobiological parameters^6^. A link between human prefrontal cortex activity and the positive affective processing of safety signals can probably be added as an explanatory mechanism for a great number of risky behaviors in patients with insufficient or inadequate sleep^7^. The plausibility of this hypothesis lay on the fact that this brain region is negatively affected by sleep loss^8^. Interestingly it has been argued that inadequate sleep and excessive alcohol consumption are directly related as a feed-forward drive to a putative allostatic load throughout the addiction^9^.

**Scheme 1:**
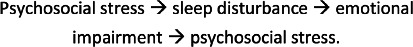
Ciclic cascate of interactive processes leading to sleep disturbance, emotional impairment, and psychosocial stress.

The end of the year transition usually favors risky behaviors associated to increased alcohol consumption and sleep deprivation, which ultimately lead to some persistent constraints regarding emotional control, including positive affect processing. Interestingly, whenever started, such hazardous behavioral pattern seems to have a trend to facilitate the persistence of a deleterious cycle impairing mood and sleep and jeopardizing wellbeing. In the pandemic critical context, this would be a plausible mechanism enhancing it toward a more severe condition (Scheme 1). However, COVID-19 pandemic restrictions imposed by governments around the world, particularly those implemented during the end-of-the-year transition, limited the extended periods of vigilance usually observed by this time of the year, as well as the alcohol consumption and other commonly reported risky behaviors. This may actually corroborate the belief on a better sleep from 31^st^ of December to 1^st^ of January among the general population.

Further, there is also little doubt that more cautious behaviors might have improved emotional processing networks related to positive thinking on this end-off-the-year transition. Still, this punctual and apparently innocent incursion of a “forced sleep hygienic behavior” on emotional processing have been promising in hampering the deleterious effect of the neurobiological and behavior related mechanisms contributing to stress persistence in the very next days as well as a prosocial enrolment^10^. It could also have been acting on different neurobehavioral networks promoting the perpetuation of positive psychophysiological processing, which ultimately lead to improved emotional affective outcomes. At the end of the day, this was probably a good start. Wasn’t it?
